# Flow behavior analysis of *Chlorella Vulgaris* microalgal biomass

**DOI:** 10.1016/j.heliyon.2019.e01845

**Published:** 2019-06-10

**Authors:** Suresh Kumar Yatirajula, Anuj Shrivastava, Vinod Kumar Saxena, Jagadeeshwar Kodavaty

**Affiliations:** a Department of Chemical Engineering, Indian Institute of Technology (Indian School of Mines), Dhanbad, Jharkhand, 826004, India; b Department of Fuel and Mineral Engineering, Indian Institute of Technology (Indian School of Mines), Dhanbad, Jharkhand, 826004, India; c Department of Chemical Engineering, UPES, Bidoli, Dehradun, 248007, India

**Keywords:** Chemical engineering, Energy, Rheology, Viscosity, Algae, Chlorella, Vulgaris, Biomass

## Abstract

The processing volume of bioengineering operations requires flow properties of algal mass for effective processing techniques. *Chlorella Vulgaris* microalgae cultured at 25 °C in Tap media under continuous illumination was considered. It showed an exponential phase of growth up to 8 days and then a stationary phase of growth from 8 days to 15 days. The rheological properties of microalgae biomass during the growth represented power law model. Microscopic analysis showed the influence of shearing on variation of algal structure from clusters to complete cell separation. The flow properties supported the microscopy analysis showing the shear thickening property at high shear rates and shear thinning nature at low shear regime. Optimal power required for the agitation of biomass based on the variations of non-Newtonian viscosity were predicted by considering the vessel geometry.

## 1 Introduction

Need for the neat and efficient energy is increasing to avoid the pollution causing emissions from the fossil fuels. For sustainable energy supply, the rapid development of renewable alternatives to fossil-derived liquid transport fuels is always significant (Rosillo-Calle, 2016). Bio-fuels like biodiesel, bioethanol and second generation biofuels from non-food feedstock have got importance concerning their carbon relief potential (Greetham et al., 2018). These technologies provide adequate fuel supply as they compete in the horticulture competence (Fernandes et al., 1991; Al-lwayzy and Yusaf, 2017; Chisti, 2007). One bio-fuel feedstock, the microalgae, has gained attention due to the increased energy production because of its potential points of interest over the maintainability issues of first and second generation feed stocks (Duygu et al., 2012).

Algae are classified based on different phylogentic groups as microalgae and seaweeds (macroalgae) (Saratale et al., 2018). Microalgae can be grown utilizing ocean and wastewater also. Most microalgae develop through photosynthesis process by utilizing daylight, CO_2_ and a couple of supplements like nitrogen and phosphorous, which are converted into biomass (Yap et al., 2016). Various kinds of microalgae are reported in the literature and exploration of these algal kinds found interesting among the researchers (Zhang et al., 2016). Most green microalgae essentially contains proteins, carbohydrates, fats and nucleic acids in different proportions. It is found that there are microalgae types that contain up to 70% of their mass by unsaturated fats (Yap et al., 2016). These kinds of microalgae can be utilized to make biodiesel by extracting the unsaturated fats present in them. It is reported that *Cholerra Vulgaris* on mixotrophic cultivation yielded higher lipid content and higher lipid productivity irrespective of culture medium, which made it potential source for the consideration of bio-diesel production. In an aeration bio-reactor with a semi-continuous mode of operation, these microalgae produced a lipid content upto 147 to 442.9 mg/lt/day, depending on the culture conditions (Pignolet et al., 2013; Sakarika and Kornaros, 2019). Protein rich biomass is obtained during the autotrophic cultivation of microalgae that are cultivated in open pond or in photo bioreactors (Silkina et al., 2017).

The *Tisochrysis isochrysis* (T-ISO) microalgal biomass concentration reported is between 0.1 g/l and 0.5 g/l in open raceway pond and between 0.5 g/l and 0.8 g/l in a photo bioreactor at a temperature of 26 °C with a flux density of 150 μmol photons m^2^ s^−1^ and in a mono specific culture made with saline sea water enriched with Walne medium and vitamins (Spolaore et al., 2006; Tzovenis et al., 2003). After dewatering the maximum biomass concentration reaches to 25% w/w solids by means of flocculation and centrifugation (Soulies et al., 2013). Further removal of water will require drying which is not economically viable. As microalgae can produce 60% or more of their dry weight in the form of oil when compared to soybeans, that contain upto 18 to 20% fat, microalgae is considered for the production of biodiesel (Spolaore et al., 2006). Use of waste water and waste land, conversion of CO_2_ into oxygen by photosynthesis, ease of growth, less pollution are the major advantages of the microalgae utilization for the production of biodiesel. *Chlorella Vulgaris* is considered in the present study as it a photosynthetic microorganism that is cultivated in the industrial sector for its lipid and high protein content that is used in the biodiesel and human food applications (Ursu et al., 2014).

The rheological properties of algal slurries are important to know for planning the upstream as well as downstream processes and for evaluating the pumping power requirement (Spolaore et al., 2006; Wileman et al., 2012). For a better microalgal biofuel generation, the rheological properties of algal slurries directly affect the energy request as it requires the capacity to process high solids algal biomass by decreasing processing volume of downstream unit operations (Fernandes et al., 1991). Concentrating and dewatering of microalgal biomass are required for lowering downstream processing volumes that reduce equipment and working expenses. One active research area geared to improve the overall microalgae biofuel production process is the rheological characterization of microalgae suspensions. The use of rheology in the microalgae production is directed towards the study of engineering design of bioreactors for optimizing the growth of microalgae and to improve some downstream processing (Soulies et al., 2013).

By considering the various factors in the biomass production, the present research is motivated towards the cultivation, harvesting and bio refinery operation. Therefore, there is a potential need for research and development to understand the fluid behavior of microalgae for effective algal growth and large scale production in an economical way. The present study aims to analyze the algal growth, rheology of algal slurries, microscopic effect of shear on the micro structure of algal cells and the effect of temperature, concentration, mechanical shearing on microalgae. This study will also enable better fundamental understanding of the rheology of algal slurries as complex fluids that can be used for process control.

## 2 Materials and methods

### 2.1 Microalgae strain and culture condition

*Chlorella Vulgaris* algal strain was obtained from microbial culture lab of environment department at IIT (ISM) Dhanbad. The culture was inoculated into 1 ml TAP media for further scale-up. This culture was inoculated into 20 ml, 50 ml, 150 ml, 500 ml and 2000 ml respectively for scale-up of culture. The species were cultivated at 25 °C under continuous irradiance of 120 mol. m^−2^ S^−1^ provided by fluorescent light bulbs (Spolaore et al., 2006). The cultures were continuously sparged with CO_2_ enriched air (2% by volume CO_2_) at a rate of 5 mLmin^−1^) (Clementi et al., 1998) in a germplasm culture room.

### 2.2 Sample preparation

To analyze the growth rate, the maximum absorbance was estimated by varying the wavelength in a UV-Vis spectrophotometer. At the wave length where the absorbance is maximum, the sample is analyzed for its absorbance at a specified time for a period of 16 days. To harvest the microalgae, the cultures were centrifuged at 3000 rpm for 10 min using REMI R-8C centrifuge after washing the cells with double distilled water for three times to remove the dissolved salts in the liquid medium. A homogeneous mixture of the final sample was achieved using a vortex mixer. This dewatered biomass was stored at 4 to 20 °C as dry microalgae sample. For the rheological analysis, before the preparation of the sample, the microalgae which was stored at 4 to 20 °C are heated to 70 °C for 20 minutes. These conditions will inactivate the cell growth while protecting the cell damage and protein denaturation. 15 g of these microalgae was mixed with 1 liter of water to have the un-sheared standard sample. To prepare the sheared samples, same amount of this mixture is placed in a mechanical agitator and mixed well at different rotational speeds for 20 minutes.

### 2.3 Rheological measurements

Rheological properties were studied by using MCR 102 Anton Paar rheometer with concentric cylinder geometry. Steady shear rate experiments were performed for samples with different concentrations obtained during the cell growth to determine the fluid behavior, by varying the shear rate and measuring the shear stress. The experiments were repeated for three times to ensure the repeatability. In measuring the geometrical characteristics, the following assumptions were made:(1) The velocity at the walls of the double wall couette cup is zero, (2) Settling is negligible as less than 1.25 (3) Microalgae are not ruptured during rheometry based on micrographs of the cells before and after measurement.

To study the effect of shear rate, the biomass sample during the rheology is examined for the microscopy. Since the rheometer does not posses the integrated microscopy facility, the sample after shearing at different shear rates, is placed immediately on the microscope for examination. It is assumed that the structural features non-equilibriate at least for some period of time.

### 2.4 Model validation and analysis

To study the non-Newtonian nature of the fluid, the basic models were tested to the data. A generalized non-Newtonian fluid which has a relation (Li and Zhang, 2003)

(1) $$\tau =K{\overset{˙}{\gamma }}^{n}$$

where *τ* is shear stress (Pa), K is the flow consistency index (Pa s^*n*^), $\overset{˙}{\gamma }$ is shear rate (s^−1^) and n is the flow behavior index which is dimensionless.

### 2.5 Power requirements for broth mixing

The power requirements in mixing the contents of broth during the growth of microalgae is an important parameter to be considered. Since the behavior of the fluid in the broth being non-Newtonian and the broth viscosity varies with the growth of microalgae, the power consumption in the broth becomes dependent on these factors. Taguchi et al. developed correlations to calculate the power required in non aerated vessels where the flow condition is turbulent and the fluid flow behavior is non-Newtonian. Using this, the power required in aerated vessels could be calculated using the correlations presented by Taguchi et al. (Taguchi and Miyamoto, 1966).

(2) $$Re^{\prime }<10\;Np=32{\left(Re^{\prime }\right)}^{-0.9}{\left(D/T\right)}^{-1.7}{\left(W/T\right)}^{0.4}\;10<Re^{\prime }<50\;Np=11{\left(Re^{\prime }\right)}^{-0.4}{\left(D/T\right)}^{-1.7}{\left(W/T\right)}^{0.5}\;Re^{\prime }>50\;Np=9{\left(Re^{\prime }\right)}^{-0.05}{\left(D/T\right)}^{-1.2}{\left(W/T\right)}^{0.9}$$

where Np is the power number, D is the diameter of the impeller, W is the width of the impeller, T is the diameter of the tank and Re' is the modified Reynolds number given by

(3) $$Re^{\prime }=\left[\frac{D^{2}N^{2-n}\rho }{0.1K}\right]{\left[\frac{n}{\left(6n+2\right)}\right]}^{n}$$

where K is the flow consistency index, n is the flow behavior index, N is the rotational speed of the impeller and *ρ* is the density of the solution. From the power number, the power required is calculated using the relation

(4) $$N_{P}=\frac{Pg_{c}}{\rho N^{3}D^{5}}$$

#### 2.5.1 Method to calculate power requirements for broth mixing

To calculate the power required for the mixing, the modified Reynold's number is calculated according to the equation 3 using the values of K and n listed in the Table 2. As the industrial bioreactors operate at a rotational speed of 10-200 rpm, a rotational speed of 60 rpm is considered for the present calculation. A vessel diameter of 1.7 m with a broth slurry height of 1.7 m are considered for the vessel design. The density of all the samples are 1015 kg per cubic meter. With the obtained modified Reynolds number, the power number is evaluated using equation 2 for various D/T and W/T values. The actual power requirements are calculated using the equation 4.

## 3 Results and discussion

### 3.1 Flow behavior during microalgae growth

**Figure 1:**
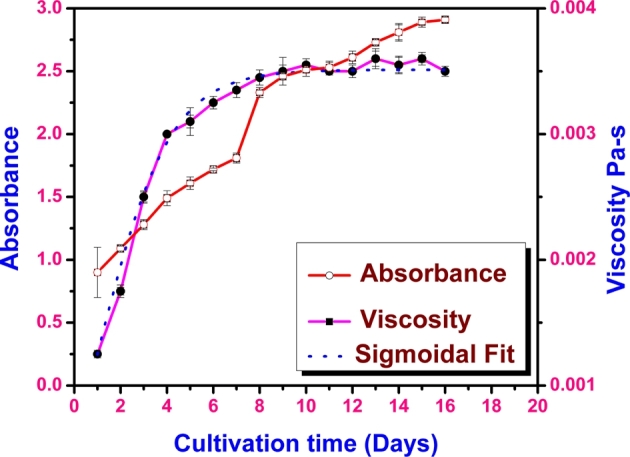
Absorbance and viscosity changes with cultivation time with a sigmoidal growth curve of microalgal slurry for the absorbance.

During the analysis of growth curve of microalgae, the maximum absorbance was found at 680 nm wavelength. The data obtained from the spectrophotometer is detailed supplementary data with the file name wavelength_data. To indicate the optimum wavelength, the data obtained from spectrophotometer at different concentrations is plotted and presented in the supplementary data with the file name optimal_wavelength_plot. The absorbance of the culture was measured every day at regular time intervals for a period of 16 days. It is observed from the results that the growth phase was observed till 8th day followed by a stationary phase, as shown in Fig. 1.

The change in the viscosity at a shear rate of 15 s^−1^ was measured for the broth during the course of the batch growth and is as shown in the Fig. 1. The Non-Newtonian behavior of the culture broth is evident from the first day of the growth as shown in Fig. 1. For a given shear rate, the shear stress increased with culture time. The growth of the microalgae is well represented by the sigmoidal growth curve and the growth, transition and plateau phases are apparent as shown in Fig. 1.

### 3.2 FTIR analysis

Cary 600 from Agilent technologies USA was used to measure the absorbance vs wave length in the range of 4000 and 800 cm^−1^. Table 1 shows the various functional groups that were present in the microalgae. The dried sample of microalgae showed the presence of amines, alkynes, cellulose, lipids, nucleic acids and polysaccharides.

**Table 1:** Main peak with the band assignment of functional group.

S. No	Main peak $\left(Cm^{-1}\right)$	Typical band assignment from the literature	Wave number range $\left(Cm^{-1}\right)$
1	3340	Water O-H Stretching	3029-3639
2	2240	C-N Stretching	2250-2220
3	2121	Alkynes C-C Stretching	2100-2260
4	1633	Cellulose v(-C=O) stretching	1583-1709
5	1417	Lipid (-CH2) stretching	1357-1423
6	1317	Nucleic Acid (>P=O) stretching	1191-1356
7	1060	Carbohydrate (C-O-C) of polysaccharides	980-1072

### 3.3 Flow properties with microscopy analysis

**Figure 2:**
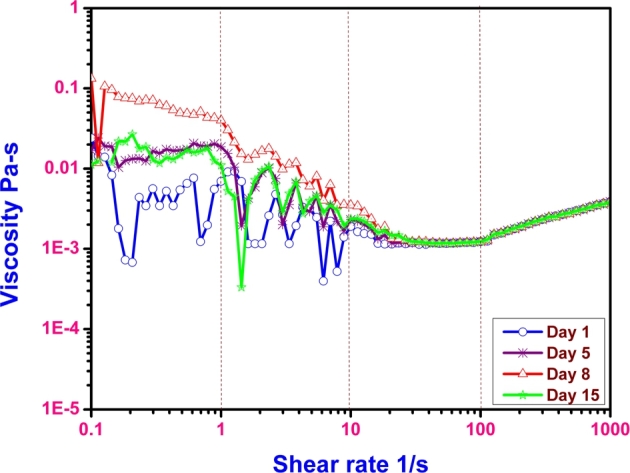
Schematic indicating the viscosity variation with shear rate at different shear regions signifying structure of biomass evident from the microscopic images.

**Figure 3:**
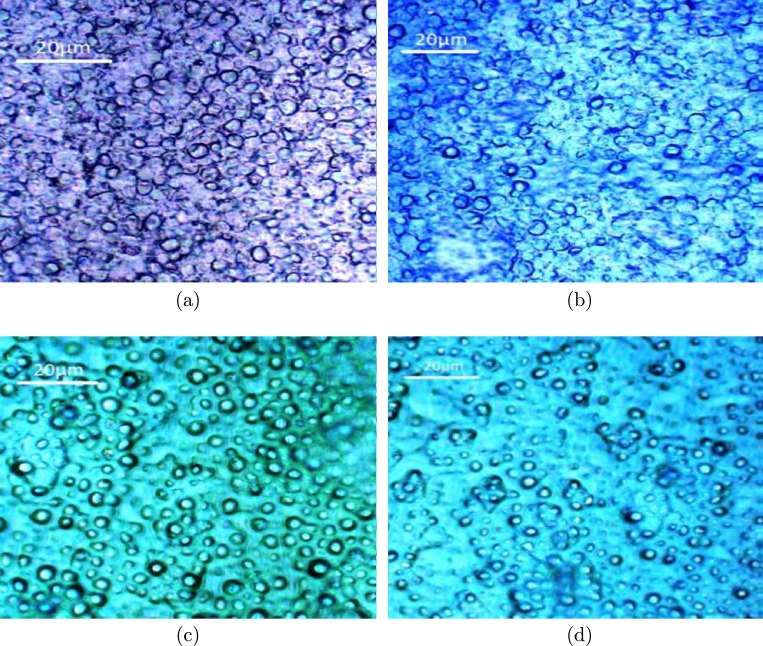
Microscopic images taken at (a) Day 1 (b) Day 5 (c) Day 8 (d) Day 15.

Biomass of microalgae solution is associated with the nutrients with various kinds of forces that forms the complex structure. Knowing the influence of shear on this complex fluid reveals the understanding of flow nature. Correlating the flow properties with the microscopy analysis gives clues of shear induced effects on these complex fluids. Fig. 2 is an example of such analysis where the flow properties and the micro structure of the fluid are compared for better understanding. Fig. 2 shows four different regimes of shear rates. The evolution of viscosity in all the regimes is evident to be different. At low shear rates, the fluid shows much of shear thinning in nature. This might be due to the breakage of hydrogen bonding associations among the cells, as the cells initially form clusters in the biomass. The microscopy image shown in Fig. 3a shows the micro structure of the regime where the shear rate ranges between 0.1 to 1 s^−1^.

With an increase in shear rate from 1 to 10 s^−1^, the aggregations of cells forms loose clusters and obstruct the flow field. The microscopic picture in the Fig. 3b shows the cluster like micro structures. In this range between 1 to 10 s^−1^, the viscosity is still shear thinning as shown in Fig. 2, but this change is limited to an order of magnitude. These changes in viscosity are comparatively less when compared to the shear range between 0.1 to 1 s^−1^, though the qualitative nature of the fluid flow being shear thinning.

In the shear rate range between 10 to 100 s^−1^, the deformation forces over come the attractive forces there by the cells align in the flow field showing no variations in viscosity, as shown in Fig. 2. The micro structure shown in Fig. 3c indicates the cell separation from cluster forming the individual cells.

At higher shear rates, in the range of 100 to 1000 s^−1^, and the flow behavior becomes slightly shear thickening as shown in Fig. 2. This argument could be supported by the micro structure as shown in Fig. 3d. The flow condition in this range of shear rate becomes shear thickening as the cell separation opposes the fluid flow field at higher shears resulting in the increase of viscosity.

During the growth of microalgae, four important periods were chosen to see the nature of the fluid. The initial period is where the microalgae growth starts, which is considered to be day 1. The second period is the middle of the growth curve, where the growth becomes steep, which is day 5. Day 8 is the third period where the growth of microalgae becomes saturated and the final phase of growth is on day 15.

**Figure 4:**
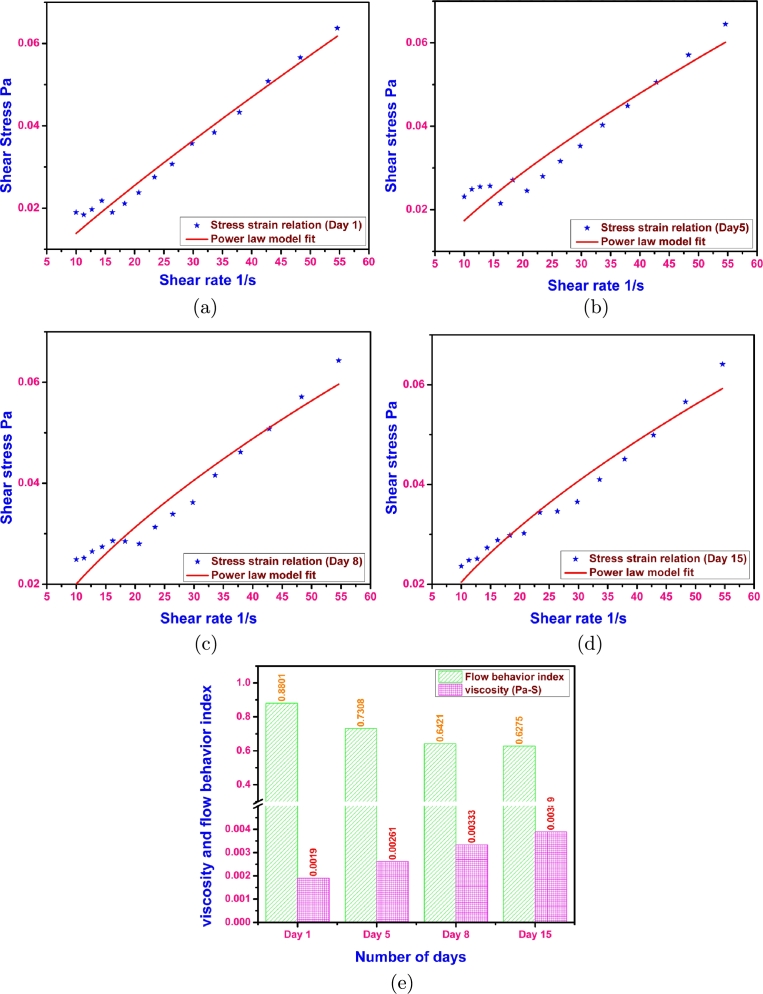
Showing the Power law model fit to the data for (a) Day 1 (b) Day 5 (c) Day 8 (d) Day 15 (e) Variation of viscosity and flow behavior index at different days during the algal growth.

Stress strain relation on these days were evaluated to fit the generalized non-Newtonian power law model as shown in Fig. 4a to 4d. It can be seen that the data is well represented by the model. The values of flow consistency index K,flow behavior index n and R^2^ values are noted down and are presented in the Table 2.

**Table 2:** Model parameters evaluated at four stages of growth.

S. No	Sample day	K (Pa s^*n*^)	n	R^2^ Value
1	Day 1	0.00183	0.8801	0.97063
2	Day 5	0.00323	0.7308	0.91247
3	Day 8	0.00457	0.6421	0.93121
4	Day 15	0.00481	0.6275	0.95325

These values are in agreement with the work done by Wileman et al. The flow behavior index values showed a value of 1 upto the concentration of 60 and for concentrations of 60% and 70%, the flow behavior index values showed 0.78 and 0.62, respectively in Wileman et al. work (Wileman et al., 2012). In the present work, the algal slurries showed a flow behavior index values ranging from 0.8801 to 0.6275 during the growth of the algal mass. This variation is because the sample preparation has been done by different methods. Wileman et al. prepared the samples by mixing the measured quantities of microalgae mass in the nutrient medium after the centrifugation and in the present work the medium is water. However, in both the cases a trend of decreasing flow behavior index value is observed. The flow behavior index values presented in our work showed similar trend.

The viscosity values evaluated at a shear rate of 15 s^−1^ in the above specified time intervals are compared with the flow behavior index. During the growth of microalgae, with increase in viscosity the flow behavior index decreases as shown in Fig. 4(e).

### 3.4 Effect of agitation speed and temperature

**Figure 5:**
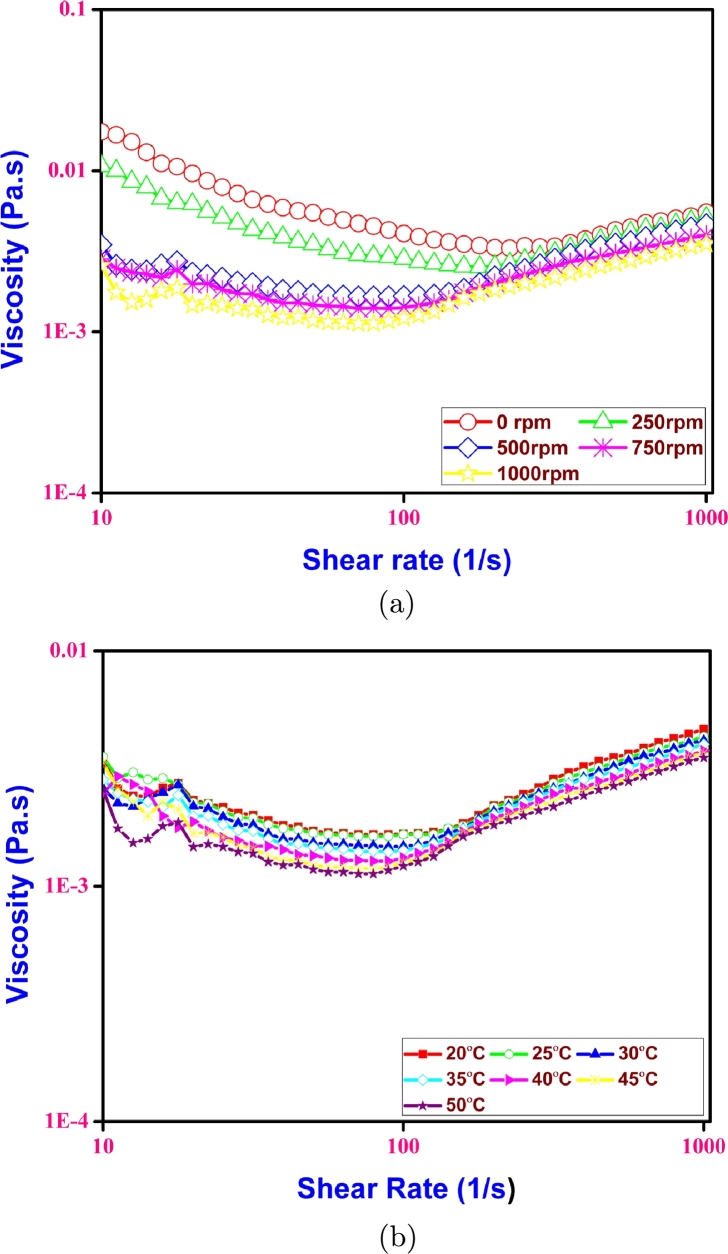
(a) Viscosity and shear rate relation at various rpms (b) Viscosity and shear rate relation at various temperatures.

Sheared samples were prepared according to the different rotational speeds with 0, 250, 500, 750 and 1000 rpm as discussed in section 2.2. These samples were subjected to stress strain analysis and the viscosity shear rate data is obtained. It can be seen from Fig. 5(a) that the viscosity at low shear regime is different for different samples. However, at high shear rates the viscosity of all the samples showed similar behavior and values. The reasons are evident as the shearing of fluid alter the structural features of the fluid as discussed in the section 3.3.

The changes in temperature could not affect the viscous properties of the biomass, as indicated in Fig. 5(b). The variation of viscosity in the broth conditions could be analyzed further.

### 3.5 Power requirement in broth agitation

**Figure 6:**
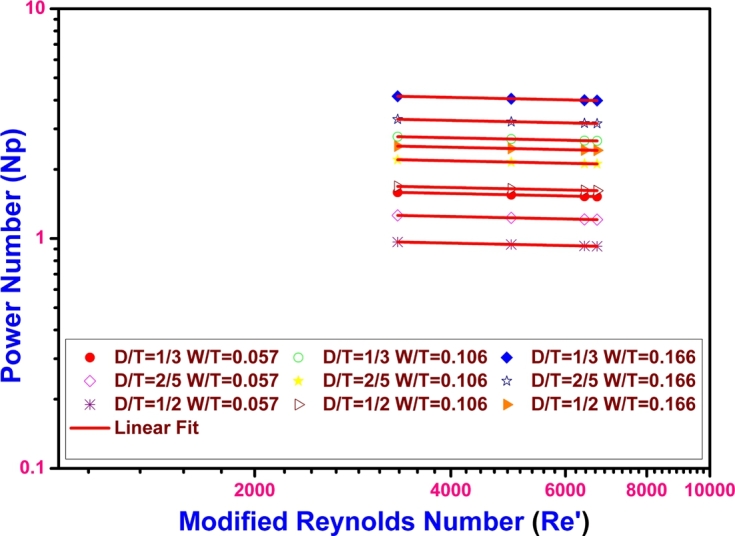
Modified Reynolds number vs Power number at different D/T and W/T values showing linear relationship.

As the present work is focused to find out the power consumption in the non-aerated vessels, the modified Reynolds number and the power numbers are calculated according the procedure mentioned in the materials and methods section. The modified Reynolds number values when plotted against the power number, resulted a linear relationship as shown in Fig. 6.

It is evident from the Fig. 6 that the power number at a set of D/T and W/T values is almost same. Though the viscosity varies during the growth of algal mass in a non-Newtonian manner, the power required largely depends on the vessel dimensions. It is calculated that the power required varies from 5.4 to 5.2 KW when the D/T is 2/5 and the W/T is 0.057. The maximum power will be utilized when the D/T is 2/5 and the W/T is 0.166 ranging the power required from 14.2 KW to 13.6 KW. In both the cases the power requirement ranges from 5.2 to 14.2 KW or 1.058 to 2.890 KW/m^3^. These values are in agreement with the values reported for mixing Xanthan fermentation broths, where the power required varied from 0.05 to 5.58 KW/m^3^ for a dual turbine mixer (Sánchez et al., 1992).

## 4 Conclusion

The present work emphasizes the experimental measurements of rheological properties of oil producing microalgae *Chlorella Vulgaris*. The microalgae grows exponentially up day 8, which shows that microalgae are nearly doubling its cells daily in this growth phase and from day 9 the stationary phase of algal growth started which proceeds upto day 16 which showed there is equal production of extra polysaccaride and equal death of cells. The viscosity of the solutions becomes non-Newtonian with varying viscosities during the growth of algal mass. The flow behavior index varied from 0.8801 to 0.6275 and the flow consistency index varied from 0.00183 to 0.00481 Pa s^*n*^, during the growth of the algal mass. The power required for agitating the contents of the broth was estimated and found to vary not with the variations of viscosity but with the bioreactor dimensions. It is found that the minimum amount of power is needed for the non-Newtonian fluid behavior in the agitated vessel when the D/T value is 2/5 and W/T value is 0.057. The power required for the agitation varies from 5.2 to 14.2 KW with varying values of bioreactor dimensions.

## Declarations

### Author contribution statement

Suresh Kumar Yatirajula, Anuj Shrivastava, Vinod Kumar Saxena, Jagadeeshwar Kodavaty: Conceived and designed the experiments; Performed the experiments; Analyzed and interpreted the data; Contributed reagents, materials, analysis tools or data; Wrote the paper.

### Funding statement

This research did not receive any specific grant from funding agencies in the public, commercial, or not-for-profit sectors.

### Competing interest statement

The authors declare no conflict of interest.

### Additional information

Supplementary content related to this article has been published online at https://doi.org/10.1016/j.heliyon.2019.e01845.

No additional information is available for this paper.

## Figures and Tables

**Fig. S1:**
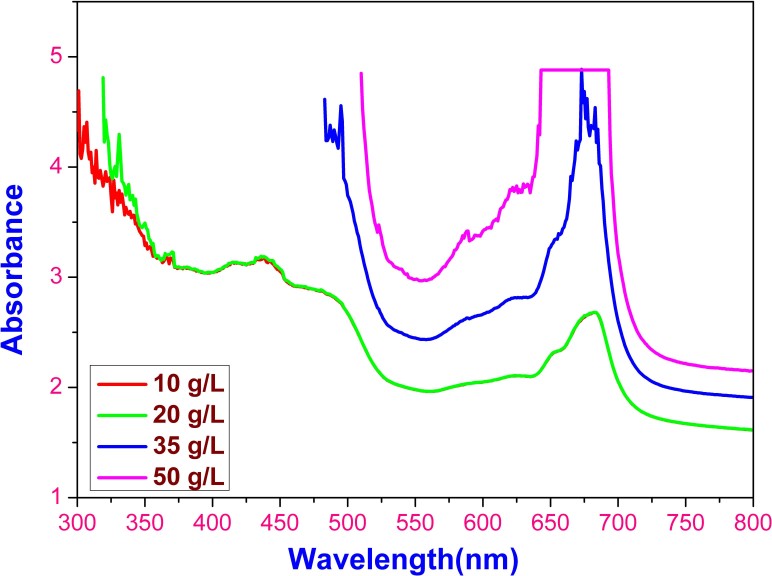
At various concentrations of microalgae samples, the absorbance and the wavelength are plotted and is found that the maximum absorbance occurs at 680 nm wavelength.

## Supplementary material

WAVELENGTH_DATA.XLSX To find the optimal wavelength, the microalgae samples at 10, 20, 35 and 50 g/L concentrations are analyzed in UV-VIS spectrophotometer by varying the wavelength and measuring its absorbance. WAVELENGTH_DATA.XLSX
